# Multiple Metastases of Parathyroid and Papillary Thyroid Carcinoma in a Female Patient Treated with Long-Term Hemodialysis

**DOI:** 10.3390/jpm13030548

**Published:** 2023-03-19

**Authors:** Julia Krupinova, Ekaterina Kim, Anna Eremkina, Lilia Urusova, Iya Voronkova, Konstantin Slaschuk, Ekaterina Dobreva, Natalia Mokrysheva

**Affiliations:** 1 Department of the Parathyroid Glands Pathology and Mineral Metabolism Disorders, Endocrinology Research Centre, Dmitriya Ulianova Street, 11, 117036 Moscow, Russia; 2 Department of Pathology, Endocrinology Research Centre, Dmitriya Ulianova Street, 11, 117036 Moscow, Russia; 3 Nuclear Medicine Department, Endocrinology Research Centre, Dmitriya Ulianova Street, 11, 117036 Moscow, Russia

**Keywords:** papillary thyroid cancer, parathyroid cancer, primary hyperparathyroidism, renal replacement therapy, case report

## Abstract

Parathyroid cancer is a rare, clinically aggressive malignancy with a prevalence of approximately 0.005% relative to all carcinoma cases and 1–5% among patients with primary hyperparathyroidism. Prognosis largely depends on the extent of the primary surgery. Non-radical surgical treatment increases the risk of local and distant metastases of the parathyroid cancer associated with limited treatment options. The combination of thyroid and parathyroid disorders has been described rather well for the general population; however, cases of parathyroid and thyroid carcinoma in the same patient are extremely rare (1 case per 3000 patients with parathyroid disorders). We present a rare clinical case of combination of parathyroid and thyroid cancers with metastases of both tumors to the neck lymph nodes in a woman with a mutation in the *MEN*1 gene (NM_130799.2): c.658T > C p.Trp220Arg (W220R), who has been exposed to radiation for 20 years before diagnosis of thyroid cancer and received renal replacement therapy with long-term hemodialysis before the diagnosis of parathyroid cancer. The patient underwent several surgeries because of metastases of the parathyroid cancer in the neck lymph nodes. Surgeons used intraoperative navigation methods (single-channel gamma detection probe, Gamma Probe 2, and fluorescence angiography with indocyanine green (ICG)) to clarify the volume of surgery. Currently, the patient is still in laboratory remission, despite the structural recurrence of tumors.

## 1. Introduction

Parathyroid cancer is a very rare malignancy (0.005% of all tumors), accounting for 1–5% of patients with primary hyperparathyroidism. Its clinical presentation is based on the symptoms of resistant hypercalcemia due to autonomous oversecretion of parathyroid hormone (PTH), which leads to bone, gastrointestinal and kidney diseases. [1,2,3,4]. Prognosis mainly depends on a volume of primary surgery [5]. Unlike benign parathyroid tumors which are most commonly treated with selective parathyroidectomy, parathyroid cancer requires en bloc resection with intact of the capsule [6]. However, reliable preoperative predictors of parathyroid cancer have not yet been identified; therefore, intra-operative recognition is obligatory. Non-radical surgical treatment increases the risk of local and distant metastases, which are resistant to radiation and chemotherapy [1,7,8]. Prophylactic lymph node dissection does not affect the overall patient’s survival and, furthermore, is associated with higher risks of postoperative complications; therefore, routine use is not recommended [9]. Etiology of parathyroid cancer includes genetic and external factors. Previous studies have found an increased risk of benign parathyroid disease in patients after radiation exposure and with concurrent thyroid disease [10]. Disease relapse occurs in 50% of cases; a third of them are regional; distant metastases are observed in 25%, most frequently to the lungs (40%) and liver (10%), and less often to bones, pleura, pericardium, pancreas and brain [11]. The 5- and 10-year disease specific survival rates are 76–85% and 49–77%, respectively [12,13]. In patients with metastatic parathyroid cancer, the 5-year survival is as low as 50% [14,15,16]. Surgery remains the most effective approach to treatment for metastases of parathyroid cancer. The treatment strategy is based on the benefit-risk ratio and implies an individual approach [1,8,17]. In retrospective studies, “aggressive” surgery for recurrent disease was associated with an 30% increase in overall survival [18,19]. The severe course of the disease is owing to hormonal activity, which leads to the development of renal failure, cardiac arrhythmias or pancreatitis due to uncontrolled hypercalcemia, in contrast to patients with non-functioning secondary lesions where overall tumor burden comes first [20,21].

Overall, the combination of thyroid and parathyroid disorders has been described rather well in general population [22]; however, cases of parathyroid and thyroid cancers in the same patient are extremely rare (1 case per 3000 patients with parathyroid disorders) [23,24,25].

We present a rare clinical case of parathyroid cancer and papillary thyroid cancer combination with metastases of both tumors to the neck lymph nodes in a woman with a mutation in the *MEN1* gene who has received renal replacement therapy for an extended time.

## 2. Material and Methods

### 2.1. Patients and Sample Collection

Informed written consent was obtained prior to inclusion in the study. This study followed the Helsinki Declaration of Principles and was approved by the Ethics Committee of our institution No.1 (2017).

Biochemical parameters of fasting blood (serum total calcium (reference interval (RI) 2.15–2.55 mmol/L), ionized calcium (RI 1.03–1.29 mmol/L), albumin (RI 34–48 g/L), phosphorus (RI 0.74–1.52 mmol/L) and creatinine (RI 50–98 mmol/L) were determined using the automatic biochemical analyzer ARCHITECT c8000 (Abbott). Intact parathyroid hormone (iPTH, (RI 15–65 pg/mL) was evaluated using the second-generation electro-chemiluminescent analyzer Cobas 6000 (Roche).

### 2.2. Imaging and Intraoperation Navigation Methods

Ultrasound examination was carried out using Toshiba (Canon) Aplio 500 with a linear probe, a frequency range of 7.0–18.0 MHz. Planar scintigraphy and SPECT/CT with ^99m^Tc-MIBI and ^131^I were performed on a General Electric Discovery NM/CT 670. The CT was conducted on a multi-detector CT scanner, Optima CT General Electric. The thickness of the sections during the study was 0.625 mm.

For intraoperative navigation were used:

- Single-channel gamma probe Gamma Finder2. The navigational activity of ^99^mTc-MIBI (150–300 MBq) is administered intravenously 60–90 min before surgery. The detection parathyroid tissue was performed, focusing on the data of preoperative topical diagnostics (ultrasound, CT, planar scintigraphy, SPECT/CT). The gamma probe is used for differential diagnosis of the neck tumors and confirmation of the complete tumor removal.
- Fluorescent angiography system SPY 3000, Novodaq. Intraoperatively, 3–4 mL of the vial with ICG was administered to the patient intravenously, followed by the injection of 10 mL 0.9% NaCl 30–60. ICG appears directly in the tissues; the detecting device must be kept at a distance of 5–20 cm from the area of interest.

### 2.3. Morphological Examination and Immunohistochemical (IHC) Staining

Histopathological diagnosis of parathyroid and thyroid tumors was established according to the WHO classification criteria (results confirmed by two independent morphologists by consensus without knowledge of the clinicopathologic information).

Sections that were 3–3.5 μm thick were made from formalin-fixed paraffin-embedded (FFPE) blocks of tumor tissue samples, which were applied to adhesive glasses (Menzel GmbH&Co KG, Bielefeld, Germany). Dewaxing and unmasking of antigens was carried out using high- and low-pH buffers (Leica, Wetzlar, Germany). Sections were stained with the anti-PTH (Cell Marque MRQ-31 1:100), parafibromin (Santa Cruz Biotechnology 2H1 1:50) and menin (Abcam ab2605 1:1000) antibodies. As an internal control of parafibromin, the endothelium of blood vessels and stroma cells was evaluated, while the external control of menin was a pancreatic neuroendocrine tumor (NET).

### 2.4. Gene Sequencing

Genomic DNA was extracted from peripheral leukocytes using PureLink1Genomic DNA Mini Kits (Thermo Scientific, Boston, MA, USA). A custom SeqCap EZ Prime Choice panel (Roche Sequencing Solutions, Indianapolis, IN, USA) targeting 22 genes (*AIP, AP2S1, CDC73, CDKN1A, CDKN1B, CDKN1C, CDKN2A, CDKN2C, CDKN2D, DICER1, GATA3, GCM2, GNA11, GNAS, MEN1, PRKAR1A, PRKCA, PTTG2, SDHA, SDHB, SDHC, SDHD*) was used for the preparation of the DNA library using NimbleGen (SeqCap EZ Prime Choice Library, Pleasanton, CA, USA) technology (Roche Sequencing Solutions, Pleasanton, CA, USA). Sequencing was performed using an Illumina MiSeq sequencer. We applied paired-end (PE) reads (minimum coverage depth × 100) strategy. Genomic coordinates refer to the latest version GRCh38.

## 3. Case Report

A 44-year-old woman with complaints of palpitation, shortness of breath, numbness, cramps and paresthesia in the upper and lower extremities was hospitalized in 2019. Palpation revealed a nodular formation on the anterior surface of the neck on the left; her height decreased by 6 cm over her lifetime. According to ultrasound scan, the thyroid nodular mass in the right lobe with a diameter of 20 mm was detected (Table 1).

Since 1984, the patient has worked at a nuclear power plant (NPP) as a chemical water treatment operator. The patient had a history of extrafascial right hemithyroidectomy and isthmectomy in 2004 (at the age of 44) due to nodular goiter. The histology report described a follicular adenoma of the right lobe of the thyroid with secondary sclerosis and calcification. The material is not available for “review”. She had an unremarkable family medical history.

At the age of 49 (in 2009), she was diagnosed with nephrosclerosis because of chronic pyelonephritis, nephrolithiasis. For the first time, a decreased renal filtration was recorded, as well as significantly increased PTH and total calcium (Table 2). In the same year, she experienced low traumatic fractures of the radius bones.

In December 2010, the patient was admitted for the first time at the Endocrinology Research Centre, Moscow. Dual-energy X-ray absorptiometry (DEXA) revealed a decrease of bone density in the hip to -4SD T-score. Over her life, the patient’s height decreased by 6 cm. Ultrasound and scintigraphy demonstrated a mass in the left lower parathyroid gland measuring 68 × 32 × 26 mm, showing a high uptake of ^99^mTc-methoxyisobutylisonitrile (^99^mTc-MIBI). Additionally, computed tomography (CT) of the chest was performed and revealed lung masses of 5.5 and 7.5 mm in the middle and lower lung fields on the left and the middle lung field on the right (the largest in S4). Differential diagnosis between post-inflammatory changes and metastases was not possible.

**Figure 1:**
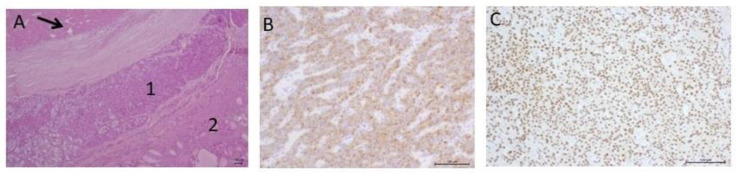
Parathyroid carcinoma. (**A**) Parathyroid carcinoma (1) with a focus of necrosis (→) invading the thyroid tissue (2); (**B**) Diffuse expression of PTH in the tumor with heterogeneous intensity in different cell pools; (**C**) Diffuse nuclear expression of parafibromin.

Intraoperatively, the surgeons noticed an additional mass (10 mm in diameter) located in the lower pole of the left thyroid lobe, which had an intimate connection with the trachea. The surgery was performed with removal of the left lower parathyroid gland and total thyroidectomy (Table 1). Fifteen minutes after the removal, there was a significant decrease in the PTH level from 2259 to 230.9 pg/mL, indicating that the operation was successful. On the third day, alfacalcidol and calcium supplementation were administered orally in high doses due to hungry bone syndrome and severe hypocalcemia. A histological examination (Table 1) diagnosed parathyroid cancer (*p*T2Nx in accordance with the 2017 AJCC TNM staging system) (Figure 1).

Over the next 3 years, there were no signs of structural or biochemical recurrence (Table 2). The lab tests showed the tendency to hypocalcemia, despite the therapy with active vitamin D metabolites and calcium carbonate. The GFR (CKD-EPI) remained stable within 10–15 mL/min/1.73 m^2^. A follow-up chest CT in 2012 showed that the dimensions and quantity of the lung lesions were unchanged (Table 1). The GFR (CKD-EPI) began to decline progressively since 2014, and in 2015 renal hemodialysis was started (three times a week, 4 h and 20 min). During 2013–2016, laboratory parameters of calcium–phosphorus metabolism were not monitored.

Six years after the operation (2016), recurrent growth of parathyroid cancer was observed on ultrasonography (Table 1), the PTH level in the needle washing liquid was more than 5000 pg/mL. Despite the recurrence of parathyroid cancer, our patient still had normal concentrations of calcium along with hyperphosphatemia (3.2 mmol/L), while her PTH level was 1713 pg/mL (15–65) (Table 2). Because of multiple abnormal lymph nodes on ultrasonography and laboratory test data, it was decided to use conservative treatment with cinacalcet (30 mg per day) and a phosphate binder (sevelamer 800 mg three times a day). With this treatment, the PTH concentration decreased to 679 pg/mL and the phosphate level to 2.28 mmol/L, while the level of calcium remained within the reference range (Table 2). The dose of cinacalcet was increased to 60 mg per day, and sevelamer to 1600 mg three times a day. Later, due to a significant decrease in BMD T-scores (to −2.5 SD in the femur, to −2.6 SD in the L1-L4 region, −4.8 SD in the radius), treatment with ibandronic acid was initiated (3 mg intravenously once in 3 months).

**Figure 2:**
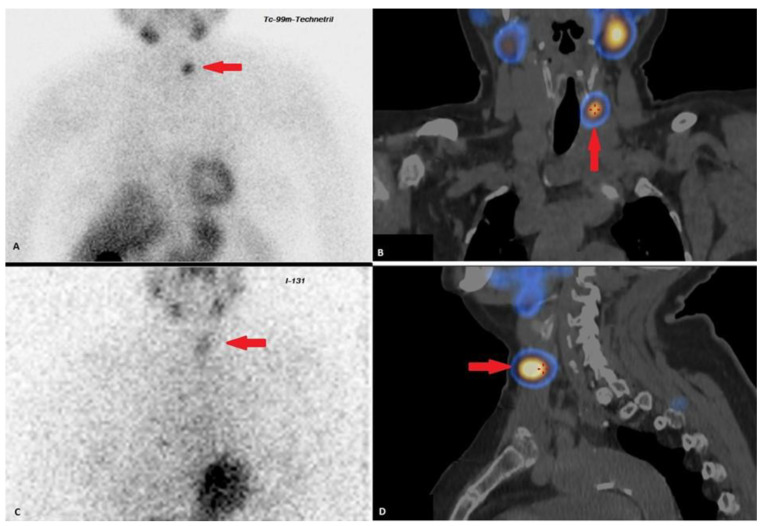
(**A**) planar scintigraphy with ^99^mTc-MIBI (←); (**B**) SPECT-CT frontal slice, high uptake of ^99^mTc-MIBI (↑); (**C**) Planar whole-body scintigraphy with I-131, (←) tissue accumulating 131I is visualized in the projection of the thyroid bed, on the left; (**D**) SPECT-CT sagittal slice, scanning was performed 90 min after the administration of 700Mbq of ^99^mTc-MIBI, (→) lesion in the left thyroid bed with high uptake of ^99^mTc-MIBI.

After 1.5 years, ultrasound revealed the progression of the tumor process, based on the size and number of lesions in the thyroid bed (Table 1). Additionally, scintigraphy with ^99^mTc-MIBI and single-photon emission computed tomography combined with CT (SPECT/CT) and the whole-body scintigraphy with 131I were performed for differential diagnosis of tumor process (Table 1, Figure 2B,C). Therefore, taking into account the results of scintigraphy and needle washing liquid measurements (PTH > 5000 pg/mL, TG 150 pg/mL), the lesion appears to be a conglomerate of thyroid and parathyroid tissue.

**Figure 3:**
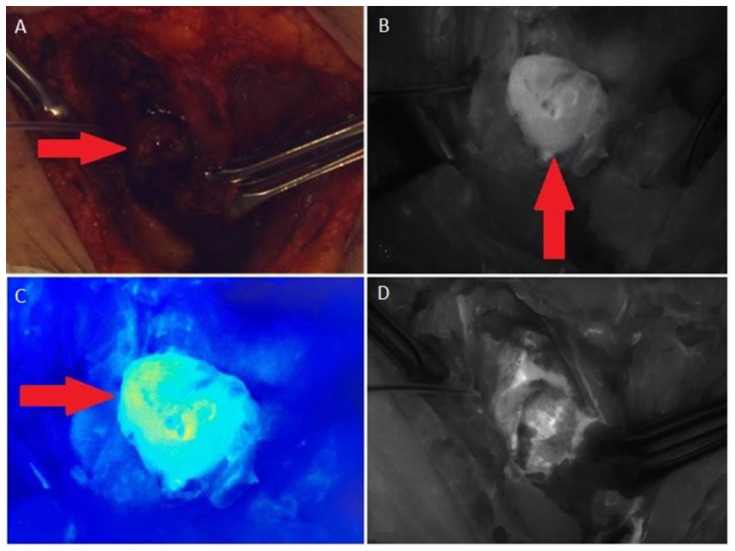
(**A**) The surgical field in the visible spectrum, the lesion (→) has been accessed; (**B**) The tumor (↑) in the infrared spectrum with the light off, 90 s after intravenous administration of ICG; (**C**) Intensive blood supply to the tumor (→), in the color spectrum; (**D**) The surgical field after tumor removed.

**Figure 4:**
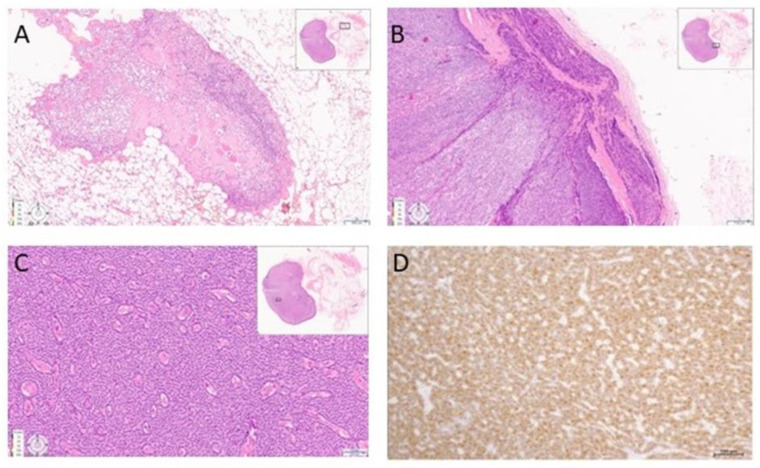
Microscopic examination of the recurrent parathyroid cancer and papillary thyroid cancer metastasis. (**A**) Metastasis of the follicular subtype of papillary thyroid cancer to a lymph node. Subtotal replacement of lymph node tissue; (**B**) Metastatic nodule of parathyroid cancer with signs of invasion; (**C**) Pattern of microscopic structure of parathyroid cancer metastasis with single mitotic figures; (**D**) Parathyroid cancer metastasis cells are positive for PTH.

A total parathyroidectomy and central lymph node dissection using intraoperative navigation methods (single-channel gamma detection probe, Gamma Probe 2, and fluorescence angiography with indocyanine green (ICG), on SPY3000 (Novadaq Technologies; Toronto, ON, Canada)) was performed in March 2019. During the surgery, the lesion in the thyroid bed on the left showing significant uptake of the isotope (^99^mTc-MIBI) and ICG was exposed (Figure 3). The histological examination revealed a piece of adipose tissue measuring 30 × 25 × 20 mm with a whitish dense nodule measuring 15 mm in diameter. There were also small gray-pink lesions with a diameter of up to 5 mm near the nodule. Microscopic examination showed a focus of parathyroid cancer in fatty tissue, and two lymph nodes with subtotal metastases of papillary thyroid cancer (Table 1). Foci of parathyroid cancer and papillary thyroid cancer were observed in surrounding skeletal muscles. The morphological features of the parathyroid cancer were similar to the histological picture of the parathyroid cancer removed in 2010 (Figure 4).

In the postoperative period, the PTH level decreased to 388 pg/mL (15–65); the albumin-corrected calcium to 1.99 mmol/L, phosphorus to 1.0 mmol/L (0.74–1.52). The patient was administered alfacalcidol 2 μg per day, calcium carbonate 1500 mg per day, and levothyroxine sodium 100 μg. This treatment led to the achievement of laboratory findings: PTH 160 pg/mL, albumin-corrected calcium 2.53 mmol/L, Ca^++^ 1.23 mmol/L.

Currently, according to the data of instrumental diagnostics (ultrasound, CT with contrast, SPECT/CT, needle washing liquid PTH measurement—more than 5000 pg/mL), a structural relapse of the disease with multiple metastases of parathyroid cancer in the lymph nodes of the neck persists. Given “non-target” sizes of lesions and the absence of uncontrolled hypercalcemia, the conservative management was continued. To clarify the etiology of primary hyperparathyroidism and parathyroid cancer, we performed parallel sequencing of genes associated with hereditary forms of disease, revealing a germline heterozygous single-nucleotide replacement in exon 4 of the *MEN1* gene (NM_130799.2): c.658T > C p.Trp220Arg W220R (CM1720809).

The family history is unremarkable. During an inpatient examination, the ultrasound and gastroscopy did not reveal any signs of NETs. Since the results of the genetic study were obtained after hospital discharges, the patient was recommended to undergo an extended examination to exclude other components of MEN1 at the place of residence. These results of additional examinations for other possible MEN1-components are not available.

**Figure 5:**
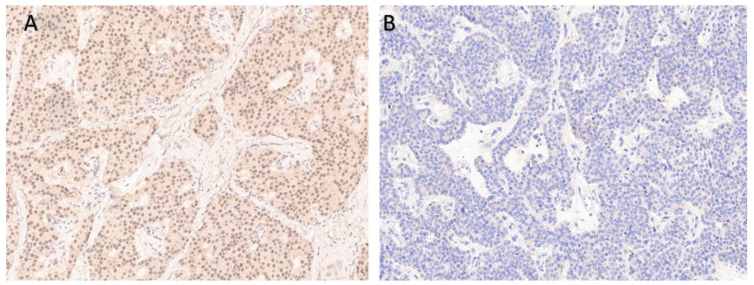
Menin IHC staining in pancreatic NET and parathyroid carcinoma. (**A**) Pancreatic tumor tissue with nuclear and cytoplasmic expression (control tissue); (**B**) Negative menin expression in parathyroid cancer.

Taking into account the germline mutation in *MEN1* gene, we had analyzed the menin expression in tumor samples by IHC (Figure 5). Parathyroid cancer was negative for menin, which was consistent with the results of the genetic study.

## 4. Discussion

Thyroid cancer is the most common endocrine malignancy, accounting for 3.4% of all cancers diagnosed annually [26]. Although well-differentiated papillary thyroid cancer may remain indolent, lymph node metastases and recurrence occurs in 50% and 20% of cases, respectively. No current biomarkers are able to predict lymph node metastasis in patients with papillary in early stage papillary thyroid cancer [27]. In the presented case, given the absence of a primary focus, according to histological examination after the second surgical treatment in 2010 and detection of metastasis in the postoperative material after the third surgery, we assume that, in 2004, there was a follicular variant of papillary thyroid cancer. The thyroid tumor manifested itself first, but the non-radical surgery in 2004 led to further progression of the disease. In addition, the diagnosis of papillary thyroid cancer was significantly delayed until histological examination after the third surgery for recurrent parathyroid cancer.

Parathyroid cancer is a rare and slowly progressing endocrine disorder. The severity of the disease is persistent hypercalcemia. However, there are no clear diagnostic criteria and treatment algorithms. The recurrence rate is up to 50%, but 10-year overall survival reaches 60–70% due to its slow-growing nature. [28]

Parathyroid cancer is typically sporadic, and much less common in the framework of hereditary syndromes. It mainly develops as part of the hyperparathyroidism-jaw tumor syndrome (HPT-JT), in which the incidence of cancer is as high as 15–37.5% [29,30,31]. Isolated clinical cases of parathyroid cancer have been described in patients with MEN-2A [32,33,34] and familial isolated primary hyperparathyroidism (FIHP) [35,36]. Parathyroid cancer in patients with MEN1 syndrome are extremely rare (0.28%) [37]; herewith, thyroid nodules are observed in more than 25% of MEN-1 patients. They are usually benign in nature and often a diagnostic finding during parathyroidectomy [38,39]. The few available studies did not confirm an association between a mutation in the MEN1 gene and papillary thyroid cancer [40].

According to the ACMG/AMP criteria [41], the W220R variant that we detected is classified as likely pathogenic (M1, PM2, PP2, PP3, PP5). The p.W220R variant (also known as c.658T > C), located in coding exon 3 of the *MEN1* gene, results from a T to C substitution at nucleotide position 658. There is no functional evidence in ClinVar for this variation. Other pathogenic amino acid substitutions have been described at position 220 of the MEN1 protein, specifically W220L in familial isolated hyperparathyroidism [42] and W220S in *MEN1* [43]. These data, as well as the high conservativeness of the amino acids in the corresponding position in other species, suggest a damaging effect of the W220R substitution on the structure and function of the protein. One of the challenges of *MEN1* genetic testing is the interpretation of missense and in-frame mutations that do not predict obvious damaging effects to the protein structure or function. The effect of mutation on the structure or function of a protein studies can be assessed by computational (in silico) predictive tools: SIFT (Sorting Intolerant From Tolerant), PolyPhen-2 (Polymorphism Phenotyping V-2), MutationTaster, MutationAssessor, and others [44]. Using Structural Analysis In Silico, Caswell et al. identified W220R in *MEN1* as pathogenic [45]. The W220R variant has not been published as a benign polymorphism, to our knowledge.

No racial, ethnic or geographical differences have been found in the prevalence of parathyroid cancer to date [1], and there is no convincing evidence of any influence of external factors on its development. Discussing this clinical case, we should remember the possible relationship between secondary hyperparathyroidism and malignant transformation of the parathyroid glands. It is suggested that an increase in reactive oxygen species occurring in metabolically hyperactive cells may subsequently lead to DNA damage in parathyroid cells [46,47]. The described patient worked at a nuclear power plant. It cannot be excluded that the radiation, together with the secondary hyperparathyroidism due to chronic kidney disease (CKD), could provoke the growth of a malignant tumor in both thyroid and a parathyroid gland. Radiation exposure can be the cause of the combination two malignancies in one patient.

The pre- and intraoperative diagnosis of parathyroid cancer is a complex task. Clarification of the localization of a malignant formation and the extent of the lesion is an important stage in the diagnosis, which affects the further treatment approach. Currently, available imaging techniques are not able to make a differential diagnosis between parathyroid cancer and adenoma.

The experience of the surgeon who can suspect a malignant tumor intraoperatively is important. Parathyroid cancers typically have a dense, often stony consistency, as well as a thick fibrous capsule. As a rule, the tumor is either soldered to or invades surrounding tissues [10,48]. According to most studies, the average size of a parathyroid cancer is 30–35 mm (<10% of such tumors measure more than 40 mm), in contrast to adenomas, whose diameter rarely exceeds 15–30 mm [1,2,10]. In the presented case, the tumor size reached 68 mm, and it was tightly attached to the surrounding structures. The diagnosis was suspected during the surgical intervention, which allowed for radical en bloc surgery. Despite en bloc resection, 6 years later, the patient developed a recurrence of parathyroid cancer, which led to a second operation.

The morphological diagnosis of malignant parathyroid tumors is still a challenge. The final diagnosis can only be made on the basis of invasive histopathologic features: angioinvasion, lymphatic invasion, perineural (or intraneural) invasion, local malignant invasion into adjacent anatomic structures, or histologically/cytologically documented metastatic disease [49]. IHC one can be used only as an additional method (biomarkers, including Ki67, PGP9.5, parafibromin (PFIB), APC, galectin-3 (GAL-3), PGP9.5 and others) [49]. To date, the morphological predictors of recurrence have not been clarified. High Ki-67 (>5) proliferation index is an important predictor of aggressive tumor’s behavior. Parathyroid carcinoma often has a Ki-67 labeling index of greater than 5%; parathyroid adenomas typically have a much lower labeling index, whilst the proliferative index of atypical parathyroid tumors may be intermediate [49,50]. None of these have enough sensitivity and specificity on its own; therefore, results need to be interpreted jointly with morphology of the tumor.

In our case pathological examination revealed extensive nodular proliferation of various cell types, stromal fibrosis and irregular borders gland. This morphological pattern can also mimic morphological features associated with parathyroid hyperplasia due to long-standing CKD or atypical parathyroid adenoma [49]. Nevertheless, the observed invasive growth of the tumor in the surrounding soft tissue and thyroid confirmed its malignant potential.

To clarify the pathogenicity of the mutation, we decided to analyze the menin expression in tumor samples. As a result, W220R variant of *MEN1* mutation was disruptive to the protein. Menin is an extremely functional protein. Pathogenic variants in the *MEN1* gene cause an autosomal dominant disorder in which patients develop neoplastic lesions in various endocrine tissues, principally in the parathyroids, pituitary and pancreas. Besides its tumor-suppressor role, menin interacts with different transcription and chromatin-modifying factors, significantly contributing to cellular signaling pathways. In addition, menin plays a role in cell proliferation, apoptosis and genome integrity [44,51]. Although a large number of mutations have been reported in *MEN1*, genetic testing complemented by IHC continues to uncover novel missense substitutions and analyze their potential pathogenicity. Currently, the patient is still in laboratory remission of the disease while having a relapse of the tumor. The multiple cervical lymph nodes with secondary changes are prognostically unfavorable for the further course of the disease and require increased alertness regarding laboratory relapse (development of severe hypercalcemia). In view of the patient’s comorbidities, choosing the optimal plan is difficult. On the one hand, there are indications for radioiodine therapy for recurrent papillary thyroid cancer. However, a separate device should be isolated, due to the need for hemodialysis sessions. Moreover, papillary thyroid cancer is less aggressive and life-threatening than persistent parathyroid cancer. On the other hand, the question of treatment of parathyroid cancer metastases remains, which obviously pose a greater threat to the patient’s life, since there are no effective regimens of chemotherapy or external beam radiation therapy (EBRT) for their treatment [52]. There are several cases of successful use of anti-PTH immunotherapy, as well as target therapy with sorafenib [53,54,55,56]. However, the decision of further therapy can only be made if the tumor progresses, or life-threatening conditions develop.

## 5. Conclusions

The presented clinical case demonstrates a rare combination of a parathyroid cancer and a papillary thyroid cancer with metastases in a patient with a history of exposure to irradiation and prolonged hemodialysis.

There are no clear preoperative diagnostic criteria for parathyroid cancer. The only curative treatment for parathyroid cancer affecting the prognosis is a complete surgical resection with margin of healthy tissue. Despite the wide possibilities of imaging techniques, they allow detection of the extent of the tumor, but not to clarify its nature. The diagnosis of parathyroid cancer should be confirmed histologically by an expert using additional methods that increase the accuracy of diagnosis.

## Figures and Tables

**Table 1:** Imaging and types of surgeries.

	Imaging	Type of Surgery	Histopathological Results
2004	Ultrasound scan—20 mm node of right thyroid lobe	Surgery 1— right hemithyroidectomy and isthmectomy	Follicular adenoma of the right lobe of the thyroid with secondary sclerosis and calcification
2010	Ultrasound: ‘a mass in the left lower parathyroid gland, 68 × 32 × 26 mm’. ^99^mTc-MIBI scintigraphy: ‘a high uptake of ^99^mTc-MIBI in the left lower parathyroid gland. Neck CT: ‘polycyclic focus of tissue with round curves of size of 59 × 35 × 30 mm between thyroid and esophagus’. Chest CT: ‘lesions 5.5 and 7.5 mm in the middle and lower lung fields on the left and the middle lung field on the right (the largest in S4). Differential diagnosis between post-inflammatory changes and metastases was not possible’	Surgery 2—selective parathyroidectomy (left lower parathyroid gland) and total thyroidectomy	Parathyroid cancer (pT2Nx) - a mixed cell tumor type consisting of chief, oxyphilic, transient and clear cells; - small cell’s groups with nuclear pleomorphism; - a mixed structure consisting of solid, trabecular and follicular structures with a tendency to nodule formation; - old and new hemorrhages, “false” cysts filled with hemorrhagic contents, a focus of necrosis, and bands of fibrosis were seen in the tumor; - capsule and thyroid tissue invasion (Figure 1A). - Immunohistochemistry (IHC): parafibromin + (tumor cell nuclei), PTH+ Ki-67—5%.
2012	Chest CT: ‘lesions with a diameter of 2–5 mm in all fields of both lungs; no change compared to the previous examinations’	-	-
2016	Ultrasound: ‘multiple hypoechoic lesions measuring up to 4–8 mm around left lobe and in the thyroid bed on both sides; altered lymph nodes suspicious for metastases’	-	-
2018–2019	Ultrasound: ‘multiple hypoechoic lesions with dimensions of 12 × 7 mm, 9 × 6 mm, 11 × 6 mm and 9 × 5 mm of the left lobe; 1.4 cm lower a new lesion 0.6 cm in diameter’ ^99^mTc-MIBI scintigraphy with SPECT/CT: ‘a round lesion with clear contours and an inhomogeneous structure, measuring 14 × 10 × 15 mm and with significant radiopharmaceutical uptake, above the jugular notch, anteriorly to the trachea, slightly to the left of the midline’. Planar whole-body scintigraphy with I-131: tissue accumulating 131I is visualized in the projection of the thyroid bed, on the left.	Surgery 3—total parathyroidectomy with the adjacent soft tissues and central lymph node dissection using intraoperative navigation methods	Metastases of parathyroid cancer (with a diameter of about 15 mm) and papillary thyroid cancer (follicular variant) to the lymph nodes with total and subtotal replacement of node tissue IHC: diffuse expression of PTH and parafibromin (Figure 1B,C), Ki-67—7%.

**Table 2:** Laboratory test results.

Time\ Parameter	PTH, pg/mL (15–65)	Albumin-Corrected Calcium, mmol/L (2.15–2.55)	Ionized Calcium, mmol/L (1.03–1.29)		Phosphorus, mmol/L (0.74–1.52)	Creatinine, μmol/L		eGFR (CKD-EPI), mL/min/1.73 m^2^
2009	2500	3.36	-	-		110	51	
october 2010	3910	3.26	1.67	1.5		287	15	
december 2010, after surgery 2	11.7	-	1.24	-		-	14	
2012	17.9	-	1.25	-		429.0	10	
2013	423	2.26	1.05	1.65		471.1	9	
2016	1713	2.47	-	3.2		Since 2015 renal replacement therapy (hemodialysis)		
2017	679	2.49		2.28				
2019, after surgery 3	388	1.99	1.3	1.0				

## Data Availability

The data that support the findings of this study are available from the corresponding author upon reasonable request.
